# Molecular Detection of Schistosome Infections with a Disposable Microfluidic Cassette

**DOI:** 10.1371/journal.pntd.0004318

**Published:** 2015-12-31

**Authors:** Jinzhao Song, Changchun Liu, Swarna Bais, Michael G. Mauk, Haim H. Bau, Robert M. Greenberg

**Affiliations:** 1 Department of Mechanical Engineering and Applied Mechanics, School of Engineering and Applied Sciences, University of Pennsylvania, Philadelphia, Pennsylvania, United States of America; 2 Department of Pathobiology, School of Veterinary Medicine, University of Pennsylvania, Philadelphia, Pennsylvania, United States of America; Queensland Institute for Medical Research, AUSTRALIA

## Abstract

Parasitic helminths such as schistosomes, as well as filarial and soil-transmitted nematodes, are estimated to infect at least a billion people worldwide, with devastating impacts on human health and economic development. Diagnosis and monitoring of infection dynamics and efficacy of treatment depend almost entirely on methods that are inaccurate, labor-intensive, and unreliable. These shortcomings are amplified and take on added significance in mass drug administration programs, where measures of effectiveness depend on accurate monitoring of treatment success (or failure), changes in disease transmission rates, and emergence of possible drug resistance. Here, we adapt isothermal molecular assays such as loop-mediated isothermal amplification (LAMP) to a simple, hand-held, custom-made field-ready microfluidic device that allows sensitive and specific detection of schistosome cell-free nucleic acids in serum and plasma (separated with a point-of-care plasma separator) from *Schistosoma mansoni*-infected mice. Cell-free *S*. *mansoni* DNA was detected with our device without prior extraction from blood. Our chip exhibits high sensitivity (~2x10^−17^ g/μL), with a positive signal for *S*. *mansoni* DNA detectable as early as one week post infection, several weeks before parasite egg production commences. These results indicate that incorporation of isothermal amplification strategies with our chips could represent a strategy for rapid, simple, low-cost diagnosis of both pre-patent and chronic schistosome infections as well as potential monitoring of treatment efficacy.

## Author Summary

Schistosomes infect hundreds of millions of people worldwide, with devastating effects on human health and economic development. One of the major challenges in treatment and control of these diseases is the accurate diagnosis of infection and monitoring of transmission dynamics. Current parasitological and serological diagnostic tests often lack sensitivity, accuracy, and reliability; more sensitive and specific molecular methods require infrastructure typically unavailable in resource-limited settings. Here, we have detected cell-free parasitic helminth DNA in serum and plasma from schistosome-infected mice using LAMP, an isothermal amplification technique, in a simple, hand-held, custom-made, field-ready microfluidic device we have developed. Schistosome DNA is detectable as early as one week following infection, long before the worms have matured and begun producing eggs. The device is small, entirely self-contained and disposable, and is readily adaptable to rapid and simple point-of-contact (POC) detection of infection. The entire process from sample collection to diagnosis can be carried out on-site, without any laboratory equipment.

## Introduction

Schistosomes and other parasitic helminths are estimated to infect at least a billion people worldwide, as well as domestic and farm animals, with substantial impacts on human health and economic development. Accurate, reliable, and inexpensive diagnostic methods are key for monitoring infection dynamics and treatment efficacy, but technological gaps in current detection methods impose significant limitations on epidemiological analysis and control strategies [1,2].

Current parasitological and serological methods for diagnosis of schistosome and other helminth infections have major limitations. For example, the Kato-Katz technique to quantify schistosome eggs is unreliable (ie, results from the same patient can vary from one day to the next) and significantly underestimates infection levels [3–5]. It also requires a fully-patent infection and so cannot detect early infections with immature worms. Antigen- and antibody-based serological tests are available, but often lack sensitivity and specificity [2,6,7]. These limitations are amplified and take on added significance when monitoring mass drug administration programs, where measures of effectiveness depend on the ability to reliably monitor treatment success (or failure), changes in disease transmission rates, and emergence of drug resistance. Indeed, the recent call by the World Health Organization for complete elimination of schistosomiasis transmission [8–10] stressed the need for sensitive and specific point-of-contact (POC) diagnostic tools, particularly in low-transmission settings [11].

Nucleic acid-based methods for detection of free parasite DNA in host blood, urine, feces, and tissues hold the promise of dramatic increases in diagnostic sensitivity and specificity for helminth infections. Technological advances and more widely available genomic data have prompted several groups to explore such approaches for a variety of parasitic infections in definitive and intermediate hosts [12–14]. Recent reports have shown that loop-mediated isothermal amplification (LAMP), a highly sensitive amplification technique that does not require thermal cycling (and its associated costs and equipment), can detect parasite DNA in a variety of platyhelminth- and nematode-infected hosts [12], including those with schistosome [15–19] infections. Like PCR, isothermal amplification methods such as LAMP are readily adaptable to real-time protocols, and can be used in conjunction with reverse transcriptase and other modifications to amplify sequences from RNA and microRNAs [20]. However, despite the clear advantages of molecular detection, deployment of these methods in POC assays has been constrained by the logistical issues and costs of the infrastructure associated with these technologies.

Here, we used animal models to adapt LAMP protocols that detect cell-free *Schistosoma mansoni* DNA in host blood to a simple, inexpensive, disposable, custom-made, field-ready microfluidic cassette for molecular diagnostics [21–23]. The cassette (hereafter called a chip) features an array of reaction chambers for isothermal amplification of nucleic acids using LAMP or reverse transcription (RT)-LAMP, and potentially other isothermal amplification methodologies. Unique to our chip is the inclusion of a nucleic acid isolation membrane within each amplification reaction chamber (supporting information S1A Fig) [22]. Nucleic acids captured on these isolation membranes serve directly as templates for amplification without a separate elution step, thus simplifying flow control. Interfacing our chip with our custom-made plasma separator [24] that combines sedimentation and filtration to separate plasma from whole blood eliminates the need for any infrastructure. Several relatively low-cost options are available for monitoring the amplification reaction, either in real time using a simple fluorescence reader, an inexpensive USB-based microscope that can be connected to a laptop computer, or, most conveniently, with a cell/smart phone camera. At their end points, reactions may be assessed by eye directly, with a reaction-diffusion column [25], or with a lateral flow strip. Thermal control for the reactions is provided either by a thin film heater (powered by a DC power source or batteries) or by an integrated, water-triggered, exothermic chemical reaction (without the use of any electrical power) [26]. This lab-on-a chip device has detected as few as 10 copies of HIV in saliva [22] and pathogenic *E*. *coli* in urine [26], and has been used for rapid genotyping of *Plasmodium*-transmitting mosquitoes [27].

In this report, we show that our device can be used for molecular detection of cell-free parasite DNA from the blood fluke *S*. *mansoni* in blood from infected animal hosts. We furthermore show that the chip can detect *S*. *mansoni* DNA in serum taken from mice as early as one week post infection, several weeks prior to the onset of parasite egg production. This work serves as proof of principle for this technology that enables sensitive detection of parasite infections using a simple, inexpensive, disposable device that can function in environments lacking even the most basic infrastructural support.

## Methods

### Ethics statement

This study was carried out in strict accordance with the recommendations in the Guide for the Care and Use of Laboratory Animals of the U.S. National Institutes of Health. Animal handling and experimental procedures were undertaken in compliance with the University of Pennsylvania's Institutional Animal Care and Use Committee (IACUC) guidelines (Animal Welfare Assurance Number: A3079-01). The IACUC approved these studies under protocol number 805244.

The human plasma samples used for diluting parasite genomic DNA came from de-identified, EDTA-anticoagulated, whole blood samples previously collected from healthy adult donors at the Hospital of the University of Pennsylvania. There were no identifiable human subjects used in these experiments, and, following review by the University of Pennsylvania Institutional Review Board (IRB), the use of this blood was designated as not meeting the criteria for human subject research, and was therefore exempted from IRB oversight (protocol #814752).

### Chip fabrication

The nucleic acid amplification cassette is shown in S1A Fig. The 46 mm x 36 mm x 3.50 mm cassette consists of three layers: a top cover made of 250 μm thick, poly [methyl methacrylate] (PMMA) film; a 3 mm thick, PMMA cassette body; and a 250 μm (0.01 inch) thick, PCR Sealers (Finnzymes) tape bottom. Both the top and bottom cover films were cut with a CO_2_ laser (Universal Laser Systems). The cassette body was milled with a precision, computer-controlled milling machine (HAAS Automation Inc.) to form three separate reactors (more are possible, if required), silica “glass fiber” membrane porous disc (“membrane”) supports, and access conduits. The top PMMA film was solvent-bonded to the cassette body with acetonitrile at room temperature. Residual solvent was removed by overnight heating at 50°C.

Each reaction chamber is connected to an inlet port and an exit port with 500 μm-wide, 200 μm-deep conduits. The reactor is 5.2 mm in length, 1.0 mm in width, and 3.0 mm in depth with a total volume of 16 μl. Each reactor is equipped with a flow-through Qiagen silica membrane (DNeasy Blood & Tissue Kit) at its entry port.

### Samples

In our experiments, we used two different types of samples: calibrated samples with known quantities of target molecules; and samples obtained from infected animals. Infected-animal samples were tested directly (whole blood) and after separating plasma or serum from the whole blood.

Calibrated samples consisted of *S*. *mansoni* genomic DNA (gDNA) spiked in human plasma. *S*. *mansoni* gDNA was obtained from the Schistosomiasis Resource Center, housed at the Biomedical Research Institute, for distribution by BEI Resources, NIAID, NIH.

Samples from infected hosts were obtained from *S*. *mansoni-*infected mice from two sources. Female Swiss Webster mice, infected with ~200 *S*. *mansoni* cercariae (NMRI strain), were provided by the Schistosome Resource Center (*S*. *mansoni*, Strain NMRI-exposed Swiss Webster mice, NR-21963). Other mice (also Swiss Webster) were infected in-house at the University of Pennsylvania by subcutaneous injection of specified numbers of cercariae (typically 100–200 cercariae per mouse). Adult worms were perfused in saline from infected mice at 7–8 weeks post infection and counted.

Infected and uninfected, negative control mice were bled either via the tail vein, the sub-mandibular and facial veins, or via cardiac puncture following euthanasia.

Serum was isolated from fresh whole blood either: (i) by centrifugation following clotting or (ii) by pipetting following clotting, but without centrifugation. To obtain crude serum without centrifugation (ii), we allowed the whole blood to clot at room temperature for approximately 30 minutes, aspirated the liquid component, and stored that material at -80°C until use. Freezing would not be necessary, however, when the test is conducted in proximity with the supernatant collection.

Plasma was separated from whole blood with our custom-made POC separator that combines sedimentation and filtration and does not require any instrumentation [24].

### Benchtop assays

In addition to primers and template, LAMP reaction mixtures (15 μL) contained 9 μL OptiGene Isothermal Master Mix ISO-100 (OptiGene, UK) and 0.5 μL EvaGreen dye (Biotium, Hayward, CA). Amplification was monitored using the BioRad Real Time PCR system at 63°C. Fluorescence data were collected at 2-min intervals over 90 min (45 cycles of 2 min each).

The LAMP assay for detection of schistosome infection in mouse plasma and serum was adapted from that used to detect the *S*. *mansoni* Sm1-7 tandem repeat sequence in *S*. *mansoni* genomic DNA and in intermediate snail hosts [16]. This highly repetitive 121 bp sequence has been estimated to comprise more than 10% of the *S*. *mansoni* genome [28]. This sequence has previously been targeted by others using PCR [29] for the amplification of *S*. *mansoni* DNA in infected host serum. The assay's sensitivity in terms of genome equivalents was determined based on a value of 0.4 pg DNA per *S*. *mansoni* haploid genome, calculated from the genome size of 364.5 megabases [30]. We based our LAMP primers on those reported previously to yield successful amplification of Sm1-7 (16), and only four primers were used because of the small size of the repeating target sequence (121 bp). The four LAMP primers (and their concentrations) are:

Sm1-7-F3: 5'-GATCTGAATCCGACCAACCG-3' (0.2 μM);

Sm1-7-B3: 5'-AACGCCCACGCTCTCGCA-3' (0.2 μM);

Sm1-7-FIP: 5'-AAATCCGTCCAGTGGTTTTTTTGAAAATCGTTGTATCTCCG-3' (1.6 μM);

Sm1-7-BIP: 5'-CCGAAACCACTGGACGGATTTTTATTTTTAATCTAAAACAAACATC-3' (1.6 μM)

### LAMP assay on the microfluidic chip

For detection of schistosome DNA in mouse serum and plasma, 20 μL serum/plasma was mixed with 20 μL lysis buffer (DNeasy Blood & Tissue Kit, Qiagen) and 20 μL ethanol, and injected into one of the amplification reactors. Nucleic acids bind to the Qiagen silica membrane in the presence of high chaotropic (denaturing) salts and low pH. Following sample injection, 50 μL of DNeasy wash buffer 1 (AW1), containing chaotropic salt and ethanol, was pipetted into the chip to remove any remaining amplification inhibitors. The silica membrane was washed with 50 μL of DNeasy wash buffer 2 containing 70% ethanol (AW2), followed by air drying for 30 s.

After binding of nucleic acids to the filter and washing, 22 μL of LAMP master mix as described for the benchtop assay, which contains all the reagents necessary for LAMP, was injected into each reaction chamber through the inlet port and membrane. Subsequently, the inlet and outlet ports were sealed using transparent tape (3M, Scotch brand cellophane tape, St. Paul, MN) to minimize evaporation during the amplification process. The chip was placed in a portable custom-made device that houses a heating system and USB-based microscope (S1 Fig) for fluorescence excitation and emission imaging. The microscope was connected to a laptop computer and the images were processed with custom MATLAB code to remove background noise and uneven illumination effects. A normalized and averaged fluorescence intensity signal for each reactor was extracted from each processed image, and from these, a typical “real-time” fluorescence amplification curve was derived. The amplicons never leave the sealed chip, decreasing the risk of cross-contamination. Additionally, the chip contains negative controls to alert against potential false positives.

## Results

### Detection of Sm1-7 by LAMP on the benchtop

**Fig 1:**
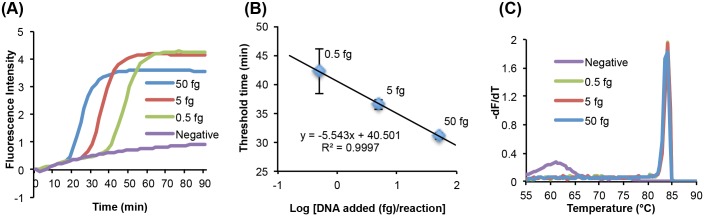
Detection of 0, 0.5, 5, and 50 fg *S*. *mansoni* Sm1-7 DNA by LAMP on the benchtop. (A) Real-time LAMP using *S*. *mansoni* Sm1-7 as the target. (B) Threshold time as a function of amount of *S*. *mansoni* gDNA (n = 3). (C). Melting curves for the reactions in A, each showing a single peak close to the predicted Tm of 84.8°C for this particular amplicon.

The Sm1-7 LAMP primers have been tested by others and exhibit high sensitivity (0.1 fg of *S*. *mansoni* genomic DNA) and specificity [16]. These primers have also been used to detect *S*. *mansoni* infection in intermediate host snails [16,17]. To examine the performance of the Sm1-7 LAMP primers with OptiGene Isothermal Master Mix, we tested the primer set against *S*. *mansoni* genomic DNA. Fig 1A depicts the real time amplification curves obtained with a bench top assay at various target DNA concentrations. Even prior to optimization, we could detect as little as 0.5 fg *S*. *mansoni* genomic DNA (with a threshold time of about 40 min). Fig 1B depicts the threshold time (n = 3) as a function of target concentration. As expected, the threshold time varies linearly with the log of the target concentration. To determine reaction specificity, we obtained the melting curves (Fig 1C). When the amplification products are present, all the curves feature sharp peaks centered at the melting temperature (Tm) of 84°C, consistent with the calculated value (using the Nearest-Neighbor module of *OligoCalc* [31]). In the absence of a target (no-template control, designated as "Negative"), there is a broad, low peak at a lower melting temperature (~62°C), presumably due to the amplification of primer-dimers. These primer-dimers do not significantly impair our detection of the target pathogen DNA.

### On-chip LAMP amplification of Sm1-7 from *S*. *mansoni* genomic DNA

**Fig 2:**
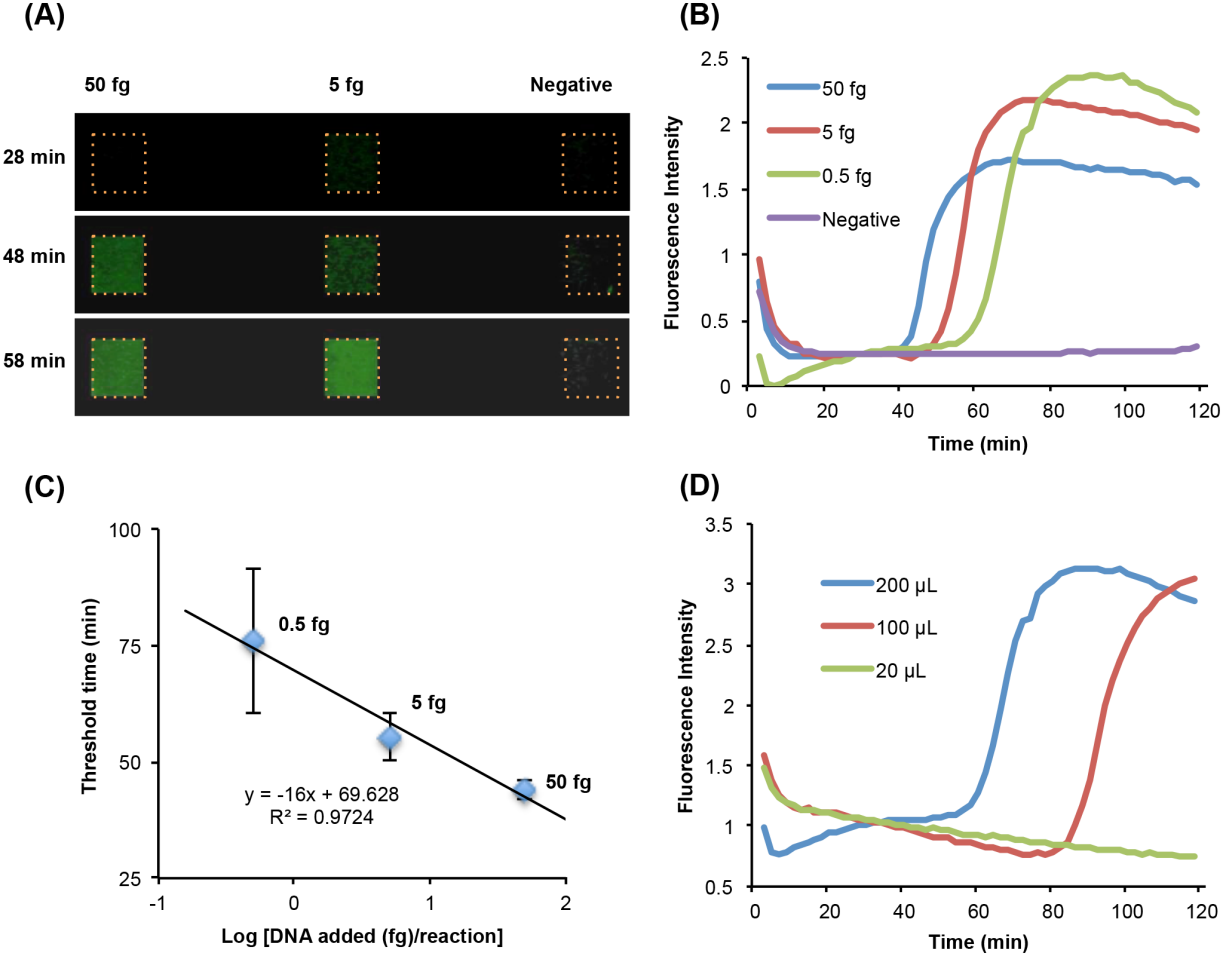
*S*. *mansoni* gDNA detection on chip with LAMP. (A) Images of fluorescence emission at t = 20, 48, and 58 min from three reaction chambers during on-chip LAMP amplification of 50 fg (left), 5 fg (middle), and 0 fg (no target control, right) *S*. *mansoni* gDNA. (B) Real time monitoring of Sm1-7 LAMP reactions with 50, 5, and 0.5 fg *S*. *mansoni* gDNA spiked in 20 μL plasma. The *S*. *mansoni* gDNA was captured on the silicon membrane and used as template. Matlab code was used to extract the averaged fluorescence intensity signals for each reactor. (C) The threshold time in B is depicted as a function of the amount of *S*. *mansoni* gDNA in 20 μL plasma, ± S.D. (n = 3). (D) Test of a sample with a very low concentration of 0.005 fg/μL *S*. *mansoni* gDNA at sample volumes of 200, 100, and 20 μL.

To examine the effectiveness of our microfluidic device for detecting schistosome infection, we spiked *S*. *mansoni* genomic DNA (gDNA) into human plasma at different concentrations. In these experiments (Fig 2), we detected and quantified the emission intensity in real time with a portable, inexpensive USB-based mini-microscope (S1B Fig). Fig 2A shows the florescent emission from three amplification reactors at various times detected with the camera (S1 Video). The reactors were loaded with 50, 5, and 0 (negative control) fg *S*. *mansoni* gDNA in plasma. Similar images were captured with a smartphone camera (S2 Fig), demonstrating that the USB microscope can be replaced with a smartphone. The emission intensity was digitized and quantified with a custom-written Matlab code. Fig 2B depicts the real-time fluorescence emission intensities (arbitrary units) detected from four reactors as functions of time. The samples consist of 50, 5, 0.5, and 0 (negative control) fg of *S*. *mansoni* gDNA spiked into 20 μL plasma. The fluorescence intensity of the negative (no target) control remains nearly level throughout the entire detection time, indicating negligible, if any, formation of primer-dimers or other non-specific products and the absence of any significant contamination. When *S*. *mansoni* gDNA is present, the signal intensity increases from the baseline to the saturation level. The higher the target concentration, the earlier the intensity curve increases above its baseline value. Fig 2C depicts the threshold time T_t_ (n = 3) as a function of the amount of *S*. *mansoni* gDNA. The threshold time T_t_ is defined as the reaction time until the fluorescent signal increases above the baseline level to ~50% of the saturation level. The figure clearly indicates that LAMP amplification of the highly repetitive Sm1-7 sequence allows the detection of far less than a single haploid *S*. *mansoni* genome equivalent (~400 fg) using relatively unsophisticated equipment. The threshold time in the chip experiments was somewhat higher than in the benchtop experiments, perhaps reflecting absorption of enzymes to the amplification reactor’s surface. Indeed, when we coated the reactor’s surface with bovine serum albumin (BSA) to reduce non-specific binding (S3 Fig), we reproduced the threshold times of the benchtop experiments (Fig 1). Despite this effect, the chip matched the benchtop sensitivity.

### Advantages of the chip over standard benchtop testing

To simplify laboratory procedures, typical benchtop assays for Sm 1–7 do not include purification, concentration, and elution steps, significantly limiting the volume of the sample that can be tested. In a typical benchtop test protocol for cell-free DNA in blood [32,33], 1 μL sample is added to the reaction mix, presumably to sufficiently dilute amplification inhibitors. For the purpose of this paper, we define protocols that add 1 μl sample to the reaction mix without prior isolation and elution as "standard" procedures. By necessity, one sacrifices test sensitivity when such a small sample volume is used. In contrast, in addition to being able to carry out our test with a simple, inexpensive, portable, battery-powered, custom-made processor (S1 Fig), our chip also offers the advantage of decoupling the sample and reaction volumes. To increase detection sensitivity, we can filter through the isolation membrane sample volumes much greater than the reaction chamber’s volume. To demonstrate this concentration function of our chip, we spiked *S*. *mansoni* gDNA into human plasma to simulate samples with a low target concentration (0.005 fg/μL). Cell-free DNA at this concentration cannot be detected when one uses a small volume such as employed in a vial-based test [32,33], but it is readily detectable in our chip simply by increasing the sample volume. Fig 2D depicts amplification curves of a sample with a target concentration of 0.005 fg/μL *S*. *mansoni* gDNA. A sample volume of 20 μL is undetectable (false negative) while sample volumes of 100 and 200 μL provide robust positive signal. Clearly, a benchtop test that utilizes a 1 μL sample will fail.

**Fig 3:**
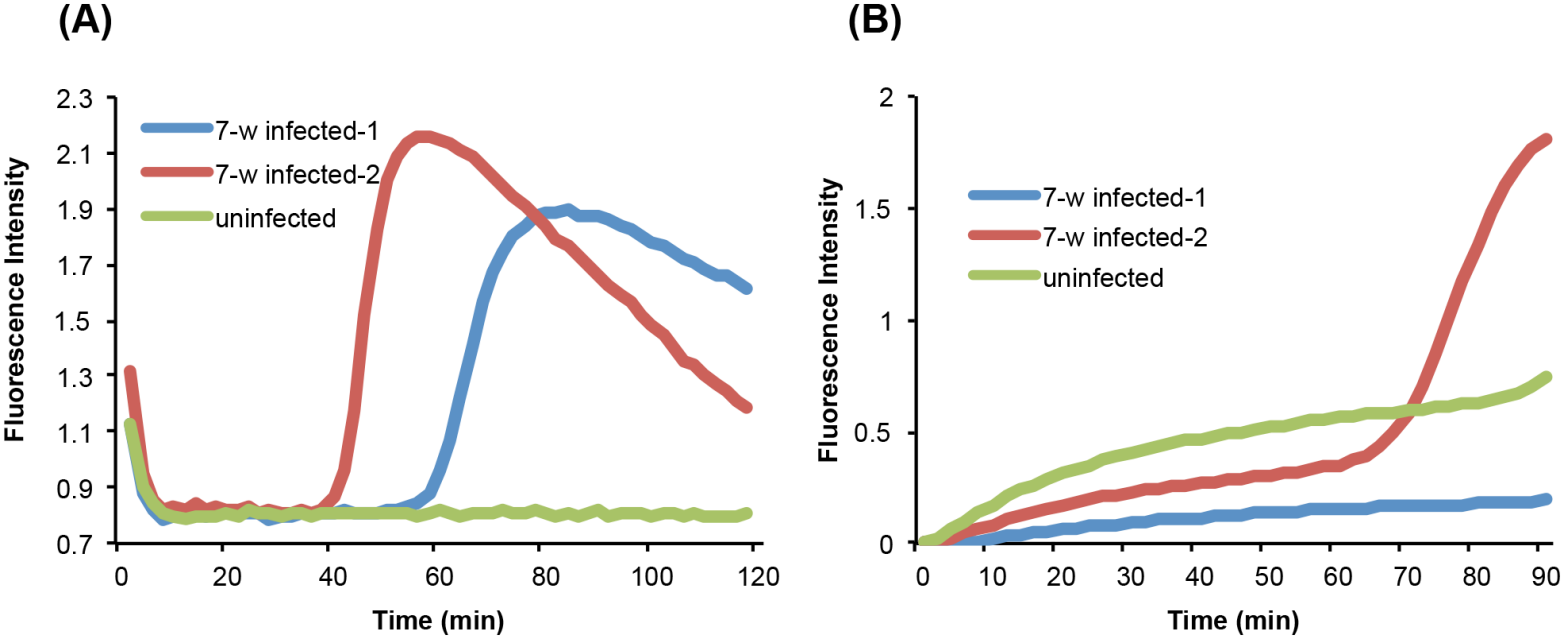
LAMP on the chip is more sensitive than benchtop LAMP (without target isolation) for detection of *S*. *mansoni* infection in mouse serum. (A) On-chip LAMP amplification using 20 μL serum from S. mansoni-infected (7 weeks post infection) and uninfected mice as templates. (B) Amplification on bench top using a standard protocol [32, 33], consisting of 1 μL serum from the same mice as in (A).

To further compare our chip (Fig 3A) with a standard benchtop test (Fig 3B), we used serum from two mice seven weeks after infection with *S*. *mansoni* cercariae, at which time the parasites have developed into mature adults that produce eggs. In the benchtop test, following recommended protocols for detection of cell-free DNA in blood [32,33], we used 1 μL of serum in the reaction mix. In contrast, we used 20 μL of serum on our chip. The chip detected a signal from both schistosome-infected murine samples while the benchtop assay had one false negative. Note also that the threshold times on the chip were significantly shorter than in the benchtop assay, indicative of the larger number of target molecules available for the on-chip reaction, consistent with the larger sample volume. In summary, the capability of our chips to concentrate nucleic acids from samples with exceedingly low concentrations of cell-free parasite gDNA and to remove contaminants and amplification inhibitors greatly enhances sensitivity and eliminates the requirement for separate DNA extraction and elution.

### On-chip detection of early *S*. *mansoni* infection in mouse serum

The Kato-Katz technique, the most commonly used diagnostic tool for schistosome infections, depends on the presence of egg-producing adult worms beginning at 5–6 weeks post infection. As an alternative, researchers have used benchtop LAMP and other amplification protocols to detect parasite DNA originating from pre-patent schistosome infections in definitive host serum [15,19] and stools [15,18]. We tested the feasibility of detecting these pre-patent infections in serum from infected mice, using our on-chip LAMP assay and 20 μL serum volume. For comparison, a few of the samples were also tested on the benchtop using the standard protocol of adding 1 μL of serum [32,33]. The results of these experiments are summarized in Table 1.

**Table 1:** The fraction of mice testing positive for *S*. *mansoni* DNA as a function of time from inoculation.

		Weeks after inoculation		
	No. of cercariae inoculated	1	3	7
Positive/examined mice on chip	200	3/3	4/4	9/9
	100	NA	3/4	5/5
Average T_t_ ± s.d. (min)	200	95 ± 3	71 ± 21	60 ± 10
	100	NA	75 ± 25*	67 ± 18

*The threshold time T_t_ of negative samples was not included in the calculation of the average.

**Fig 4:**
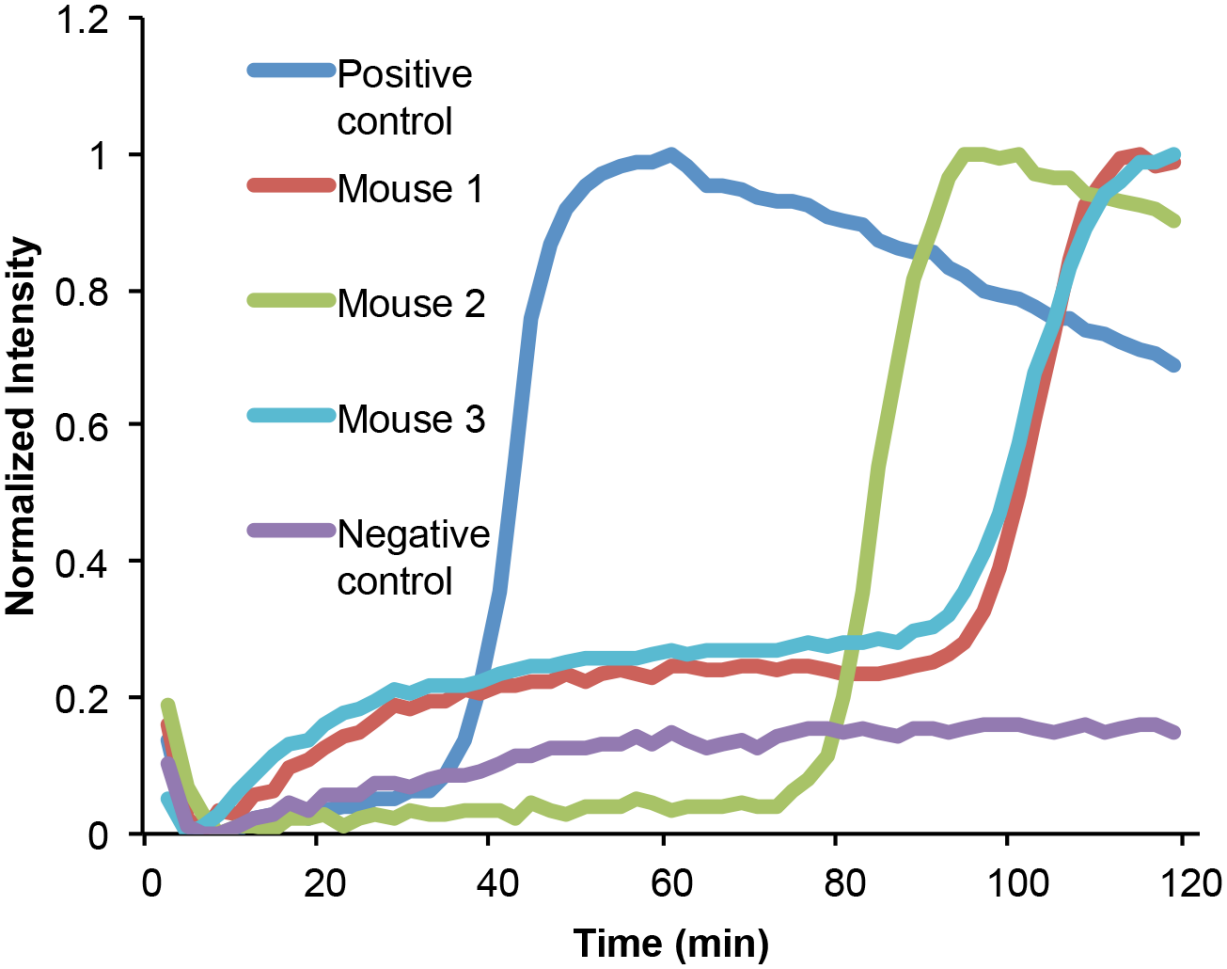
On-chip detection of early *S*. *mansoni* infection in mouse serum at 1 week following infection. The mice were infected with ~200 cercariae. Positive control was established by spiking 50 fg *S*. *mansoni* gDNA into 20 μL plasma.

Similar to previous results from LAMP benchtop assays [15,18,19], we observe a positive Sm1-7 signal in serum from three mice as early as one week following inoculation with 200 cercariae, several weeks prior to onset of parasite egg production. Fig 4 depicts the corresponding real time amplification curves at one week post inoculation. Differences in the threshold time indicate variations in the DNA concentrations, most likely resulting from variations in the number and survival of infected parasites. As the time from infection increases, so does the DNA concentration as reflected by the declining threshold time (see S4 Fig for additional data). Although we did not monitor the presence of SM1-7 in mouse blood beyond 7–8 weeks after inoculation, we expect that once the infection has reached a chronic state, the DNA concentration will achieve a steady state, consistent with reports of continued molecular detection of cell-free schistosome DNA in chronic infections [15,29,34,35].

We also tested serum from mice infected with lower numbers of cercariae (100 cercariae, average adult worm recovery = 21 ± 16.9). As Table 1 and S4B Fig show, three of the four infected samples tested exhibit positive signals at three weeks following infection; by 7 weeks, serum from 5 out of 5 mice produced a relatively strong positive signal (S4C Fig); indeed, a mouse with only 4 adult worms recovered showed a positive signal at 7 weeks.

We also repeated a few of the tests on the benchtop, using the standard 1 μL sample volume [32,33] (S4D Fig). Not surprisingly, given the smaller sample volumes used in the benchtop experiments, the benchtop sensitivity was much inferior to that of our chip. The benchtop assay exhibited, respectively, 0/3 and 2/3 positives at one and three weeks following infection with 200 cercariae.

Moreover, our chip can provide semi-quantitative data on DNA concentration. Based on the threshold time T_t_ and our standard curve obtained with *S*. *mansoni* gDNA (Fig 2C), we estimate that the DNA concentrations of *S*. *mansoni* gDNA averaged, respectively, 0.025, 0.04, and 0.25 fg/μL one, three, and seven weeks post infection.

In summary, we have demonstrated that the chip-based assay readily detects early, prepatent infection and has a sufficient dynamic range to detect and monitor DNA concentrations in early and most likely in chronic infections as well. Moreover, the semi-quantitative nature of the assay should allow one to monitor disease progression and perhaps the effectiveness of therapy [29], particularly in mass drug administration programs.

### On-chip detection of *S*. *mansoni* DNA in plasma and serum separated from whole blood without centrifugation

In the tests described earlier, we used the standard laboratory procedure of centrifugation to separate serum from whole blood. Centrifugation is, however, incompatible with point-of-care diagnostics. To eliminate the need for centrifugation, we have recently developed a unique, inexpensive plasma separator [24] that combines sedimentation and filtration to separate relatively large volumes of plasma from whole blood without any instrumentation. In the first set of the experiments reported in this section, we used our separator to separate plasma from whole blood prior to introducing the sample into the chip.

**Fig 5:**
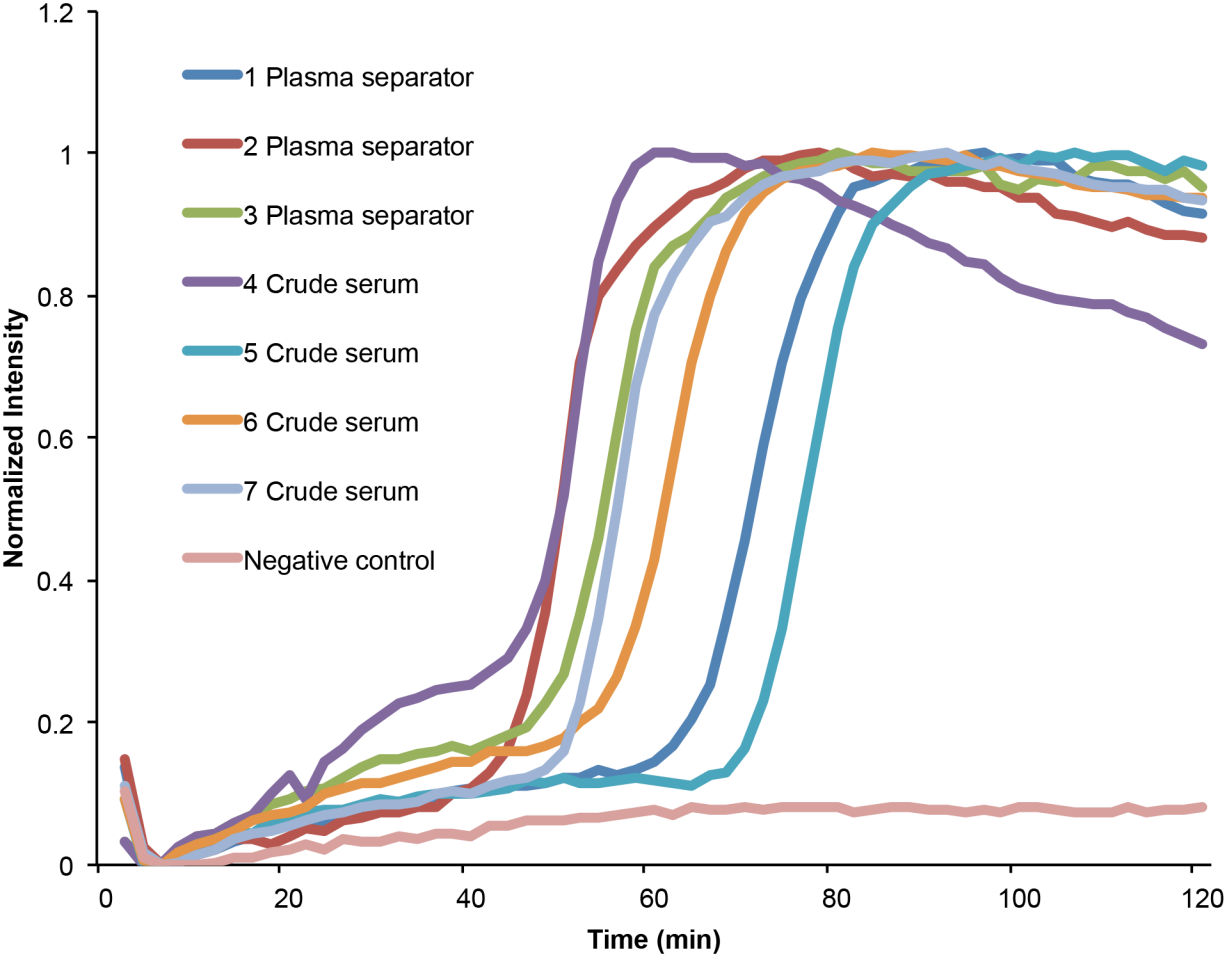
On-chip detection of *S*. *mansoni* DNA in plasma separated and serum from whole blood without centrifugation. Curves 1, 2, and 3 correspond to plasma separated with our plasma separator. Curves 4, 5, 6, and 7 show Sm1-7 amplification from crude serum separated from clotted whole blood without centrifugation. All samples were obtained from mice at 7 weeks post infection.

Alternatively, one can aspirate the liquid component from whole blood that has been allowed to clot (without centrifugation) to provide crude serum in a manner consistent with a point-of-care approach. We used plasma and serum separated with these two approaches in our on-chip LAMP assay, and obtained results similar to the ones described in the earlier sections. For example, Fig 5 depicts amplification curves of mouse samples (obtained 7 weeks after infection with 200 cercariae). The figure shows that *S*. *mansoni* DNA can be detected as readily in either plasma or serum (T_t_ ± s.d. = 61 ± 10, n = 7) obtained with these POC methods as in plasma or serum separated by centrifugation (T_t_ ± s.d. = 63 ± 16, n = 7).

## Discussion

A major challenge in treatment, control, and monitoring of neglected tropical diseases is to develop highly sensitive and accurate diagnostic methods as adjuncts or replacements for current labor-intensive and unreliable procedures [1,2]. Sensitive and specific methods for detecting early, pre-patent schistosome infections may also help prevent irreversible pathological reactions induced by eggs, and might help monitor and possibly determine the basis for treatment failures (praziquantel is relatively ineffective against immature worms). Since schistosomiasis is particularly prevalent in low-resource settings, lacking appropriate laboratory facilities, inexpensive tools for point-of-care diagnostics that do not require centralized laboratory support are highly desirable. It is not surprising, therefore, that various groups are developing and evaluating point-of-care diagnostic tools that rely on circulating antigens [36,37] and electrochemical detection in a sandwich assay format of RNA derived from parasite eggs in urine [38]. All these assays suffer, however, from low sensitivity and occasionally lack of specificity.

Molecular diagnostic tests are widely accepted as the gold standard since they provide high sensitivity and specificity, are capable of distinguishing between various species and strains of the pathogen, and are relatively easy to adapt to new threats. Since conventional molecular diagnostics requires elaborate sample processing, expensive equipment, and highly trained personnel, molecular diagnostics has been mostly confined to centralized laboratories.

To overcome some of the shortcomings of traditional molecular diagnostic techniques, we have developed a platform that simplifies sample processing and flow control and enables one to carry out all unit operations at the point of contact without reliance on expensive laboratory equipment. Although our technology would benefit from further improvements to facilitate automated, seamless operation, it can be deployed in its current state at the point of care to satisfy urgent needs. Once fully developed, this technology could be implemented for diagnostics and epidemiological monitoring in endemic settings, where sensitivity, reliability, cost, and convenience are paramount [39]. Parasitological diagnosis methods such as the Kato-Katz method for detecting eggs in stool are useful, but suffer from poor sensitivity and reliability [3,4], and are laborious. The POC circulating cathodic antigen (CCA) test on urine samples has proven to be quite useful, but suffers from reduced sensitivity in areas of lower endemicity as well as other challenges [12]. Molecular tests offer outstanding sensitivity and specificity, but are seldom used in the field due to the need for expensive equipment and highly-trained personnel [39]. In contrast, our LAMP-based system would require essentially no equipment or special training. Finally, the estimate for per-person costs of current diagnostic tests ranges from ~US$7.00 for a single Kato-Katz test, US$17.50 for a more reliable triplicate Kato-Katz test, and ~US$7.25 for the CCA test [40]. Although it is not yet possible to estimate the cost of our chip, the materials and reagents are not particularly expensive, and we would expect the per-person costs to be competitive with the other less effective tests.

**Fig 6:**
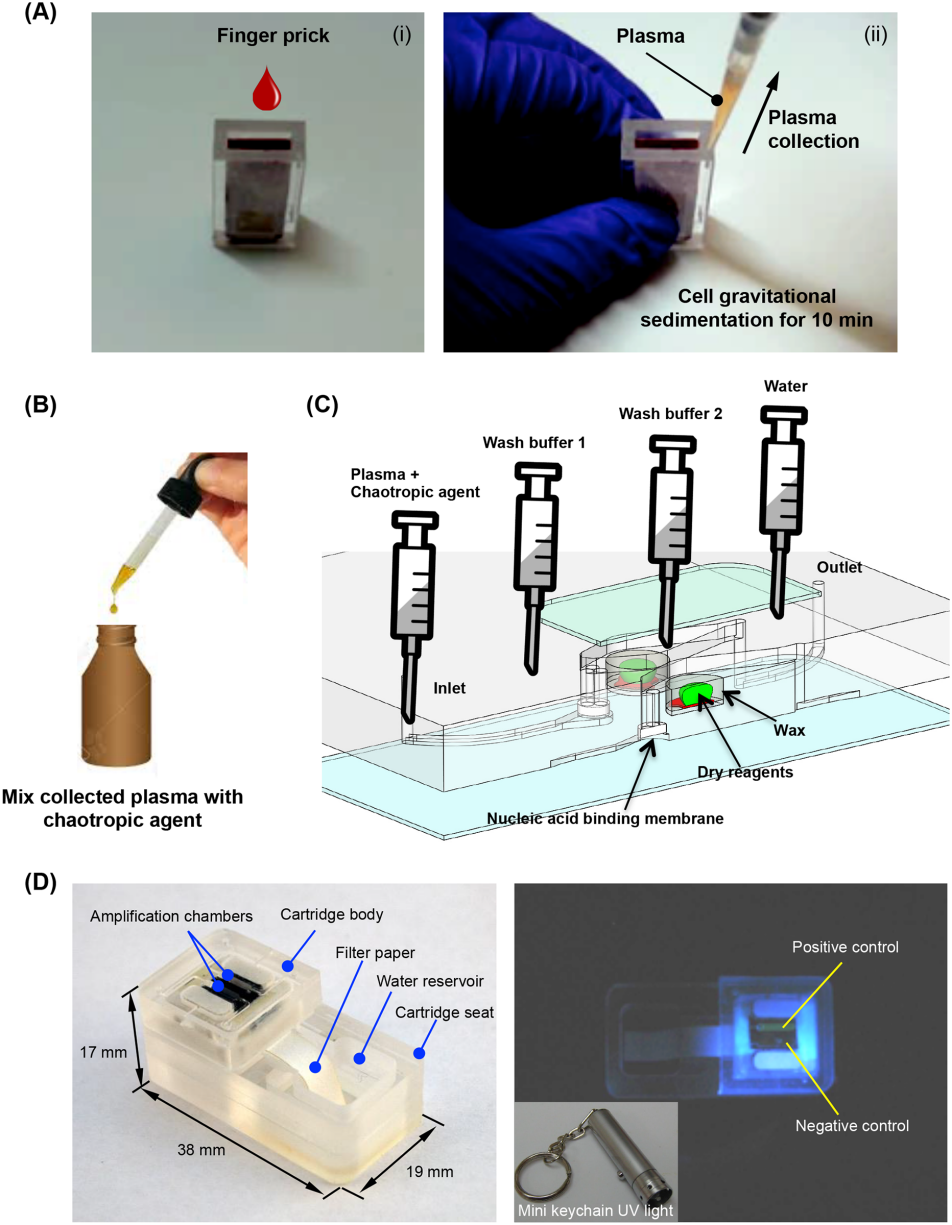
On-site, instrument-free molecular detection of parasitic helminth infections in blood samples. (A) A drop of blood, obtained from a finger stick, is dropped into the collection chamber of a POC plasma separator that combines sedimentation with filtration to separate plasma from whole blood [24]. The separated plasma is retrieved with a syringe, pipette, or dropper. (B) The plasma is mixed with chaotropic agents, and (C) inserted into the chip. The chip is preloaded with dry reagents encapsulated in paraffin [43]. As the plasma/chaotropic agent mixture filters through the isolation membrane, nucleic acids bind to the membrane and are subsequently washed. The reaction chamber is then filled with water [22]. When the chamber is heated to its operating temperature, the wax encapsulation melts and the reagents are hydrated. (D) The chip is heated to 65°C either with a resistance heater or from an exothermic reaction [26], in which water reacts with a magnesium alloy to produce heat. When an exothermic reaction is used to incubate the LAMP reaction, the temperature is regulated with a phase change material that melts at the amplification reaction temperature. A UV light source (right, inset) excites fluorescence (right) and the emission is detected either by eye or with a smartphone camera (S2 Fig). The phone can generate the amplification curve, estimate the threshold time, and communicate the results to data repositories.

Fig 6 details the sequence of operations that are needed to detect cell-free, parasite nucleic acids in blood samples. Although LAMP is reported to be less susceptible than PCR to inhibition by compounds in whole blood such as heme-protein complexes [41], it may still be adversely impacted by these inhibitors [42]. Therefore, the first step includes separation of plasma from whole blood, which can be accomplished using our POC plasma separator with a pipette or dropper and without a centrifuge. A single drop of blood from a finger stick should be sufficient. Alternatively, as we have shown (Fig 5), serum separated from whole blood without centrifugation can also be used directly in this system.

The plasma/serum is then mixed with nucleic acid binding agents and filtered through our unique, multifunctional chip that combines nucleic acid isolation, concentration, and purification. During this process, the nucleic acids bind to the isolation membrane that is installed at the inlet to our amplification reactor and the filtrate is discharged to waste. The nucleic acid isolation is carried out as part of the sample introduction into the chip, and does not require a separate operation (i.e., a spin column) nor elution. This is followed with wash steps, which require only pipetting operations and no instrumentation. Next, the reaction chamber is filled with water. In the experiments described here, we pipetted the various reagents needed for LAMP amplification into the reactor. In actual field use, we expect the reaction mixes to be pre-stored in the reaction chambers. Sample application and removal could be accomplished with an inexpensive, disposable calibrated dropper as opposed to syringes or pipette tips. To prevent the sample and wash solutions from washing away the reagents, we will encapsulate the reagents in paraffin. In separate work, we have demonstrated that this storage method provides at least six months (and likely much more) of refrigeration-free shelf life without any deterioration in the reagents’ effectiveness [43]. Once the chip is heated to its operating temperature, the paraffin melts, moves out of the way, the reagents are hydrated, and are available for the amplification reaction. The stored reagents include intercalating dye that allows us to monitor the progress of the amplification process in real time. Various reviews (i.e., [44]) have emphasized the need for these types of advances in sample preparation, processing, and detection.

In this paper, for heating and fluorescent emission detection, we used an inexpensive, home-made, battery-powered processor (S1 Fig). In separate work [26], we demonstrated that heating can be provided with an exothermic reaction and temperature can be regulated with phase change material, eliminating the need for electric power. Similarly, as we have shown (S2 Fig), a smartphone can be deployed to monitor fluorescent emission intensity [45], greatly reducing the cost of the system. Since smartphones are ubiquitous even in resource-poor countries, one can assume their availability.

The core of our platform is the multifunctional amplification reactor that combines a number of unit operations such as nucleic acid isolation, concentration, purification, and amplification that are typically carried out in the laboratory separately. An added benefit of our method is that the sample volume is decoupled from the amplification reaction volume. In other words, we can use sample volumes that are much greater than the reaction volume, improving effective assay sensitivity. Indeed, in this work, we have demonstrated sensitivity of ~25 ag/μL (2.5 x 10^−17^ g/μL) for schistosome gDNA (based on detection of 0.5 fg *S*. *mansoni* DNA in a 20 μL sample). This sensitivity is two orders of magnitude higher than that of benchtop PCR protocols without sample concentration (which are limited to small sample volumes) and many million-fold better than methods that do not use amplification. This high sensitivity facilitates early detection, within a few days of infection in asymptomatic patients, well prior to the production of eggs. The system can also be used to monitor infection progression, chronic state, and perhaps the effectiveness of therapies [12,30]. Indeed, various groups have demonstrated that cell-free schistosome DNA is detectable in chronic human infections [15,29,34,35] and, in animal models, at >100 days post infection [15,29,35]. This high sensitivity also suggests the feasibility, which we did not explore here, of detecting cell-free DNA in other body fluids, such as saliva and urine [5], which are less intrusive to collect than blood. Furthermore, our method can readily be modified for other parasitic helminth infections and to detect RNA and micro RNAs, with further improvements in sensitivity and potential monitoring of infection stage or severity.

Simple benchtop assays [32,33], unlike our chip, do not incorporate nucleic acid isolation and elution, and the total volume of the sample and the reaction mix are restricted, typically to 25 μL. These characteristics limit one to small sample volumes (typically, one or a few μL) to assure sufficient dilution of inhibitors and contaminants and appropriate concentrations of the various ingredients needed for the amplification process. In contrast, in our chip, only the volume of the reaction mix is fixed. The sample volume is decoupled from the reaction volume, allowing the use of relatively large samples without increasing the concentration of inhibitors that adversely affect amplification efficiency. Since nucleic acid isolation is part of the sample introduction process in our chip, and no separate elution is required, our chip provides better performance than simple benchtop assays, without any added complexity. Since amplicons never leave the sealed chip, cross contamination is unlikely to be a significant problem, and can be further monitored with appropriate negative controls built into the chip.

As we have demonstrated here, our chip can house an array of amplification reactors, all of which can be monitored concurrently with a single camera. This enables us to use one or more reactors for positive control and calibration. For example, in a positive control reactor, we could store a known quantity of target DNA and monitor its amplification to verify that the amplification process operates correctly as well as obtain the threshold times of known quantities of DNA to construct a calibration line. Multiple reactors can also be used to detect concurrent infections and possibly drug-resistant parasites once appropriate markers are defined.

## Supporting Information

S1 Fig **(A) A photograph and a schematic depiction of a POC chip with three reaction chambers.** Nucleic acid capture, washing, amplification, and detection are all carried out in a single chamber that houses the nucleic acid binding membrane. (B) The custom-made, portable processor for nucleic acid isothermal amplification and detection that we used in our experiments. The USB microscope can be replaced with a smartphone camera.
(TIF)

S2 Fig Smartphone detection of on-chip LAMP amplification of SM1-7 from *S*. *mansoni* gDNA. Images of fluorescence emission at t = 65 min from three reaction chambers during on-chip LAMP amplification of SM1-7 from 50 fg (top), 5 fg (middle), and 0 fg (no target control, bottom) *S*. *mansoni* gDNA. The image was taken using a Samsung Galaxy S3 smartphone. Excitation light source is from the flashlight of the smartphone with an added emission optical filter. Further experiment details are described in [44].
(TIF)

S3 Fig Investigation of the effect of the absorption of enzymes to the amplification reactor’s surface. The chamber surface was coated with 10% BSA, left to dry overnight, and then washed. The chamber was then used to amplify 50 fg *S*. *mansoni* gDNA in serum. The threshold times in the BSA-coated and uncoated chambers were, respectively, 27 ± 1.4 and 41 ± 1.4 min (n = 2). We hypothesize that the BSA coating reduced enzyme unspecific binding to surfaces, thereby increasing amplification efficiency. The BSA-coated reactor performed similarly to the benchtop.
(TIF)

S4 Fig On-chip detection of early *S*. *mansoni* infection in mouse serum. (A) Mice infected with ~200 cercariae, tested 3 weeks post infection. (B) Mice infected with ~100 cercariae, tested 3 weeks post infection. (C) Mice infected with ~100 cercariae tested 7 weeks post infection. The adult worm recovery number for mice 1, 2, 3, 4, and 5 in C were, respectively, 17, 49, 13, 22, and 4 parasites (adult worms were perfused from mice at ~7 weeks and counted). The positive control consists of 50 fg of *S*. *mansoni* gDNA spiked in 20 μL plasma. (D) Amplification on benchtop using 1 μL serum from the same mice as in (A) and Fig 4. Curves 1, 2, 3, and 4 correspond to 3-week-infected mice. Curves 5, 6, and 7 correspond to 1-week infected mice. The experimental conditions are the same as in Fig 3B.
(TIF)

S1 Video USB-microscope monitoring of the fluorescent emission from three amplification chambers containing 50 fg, 5 fg, and 0 (negative control) target DNA. (AVI)
