# Supplementary figures and images for: Molecular Detection of Schistosome Infections with a Disposable Microfluidic Cassette
Source: PLoS Negl Trop Dis. 2015 Dec 31;9(12):e0004318. doi: 10.1371/journal.pntd.0004318 (PMC4700990; doi:10.1371/journal.pntd.0004318)

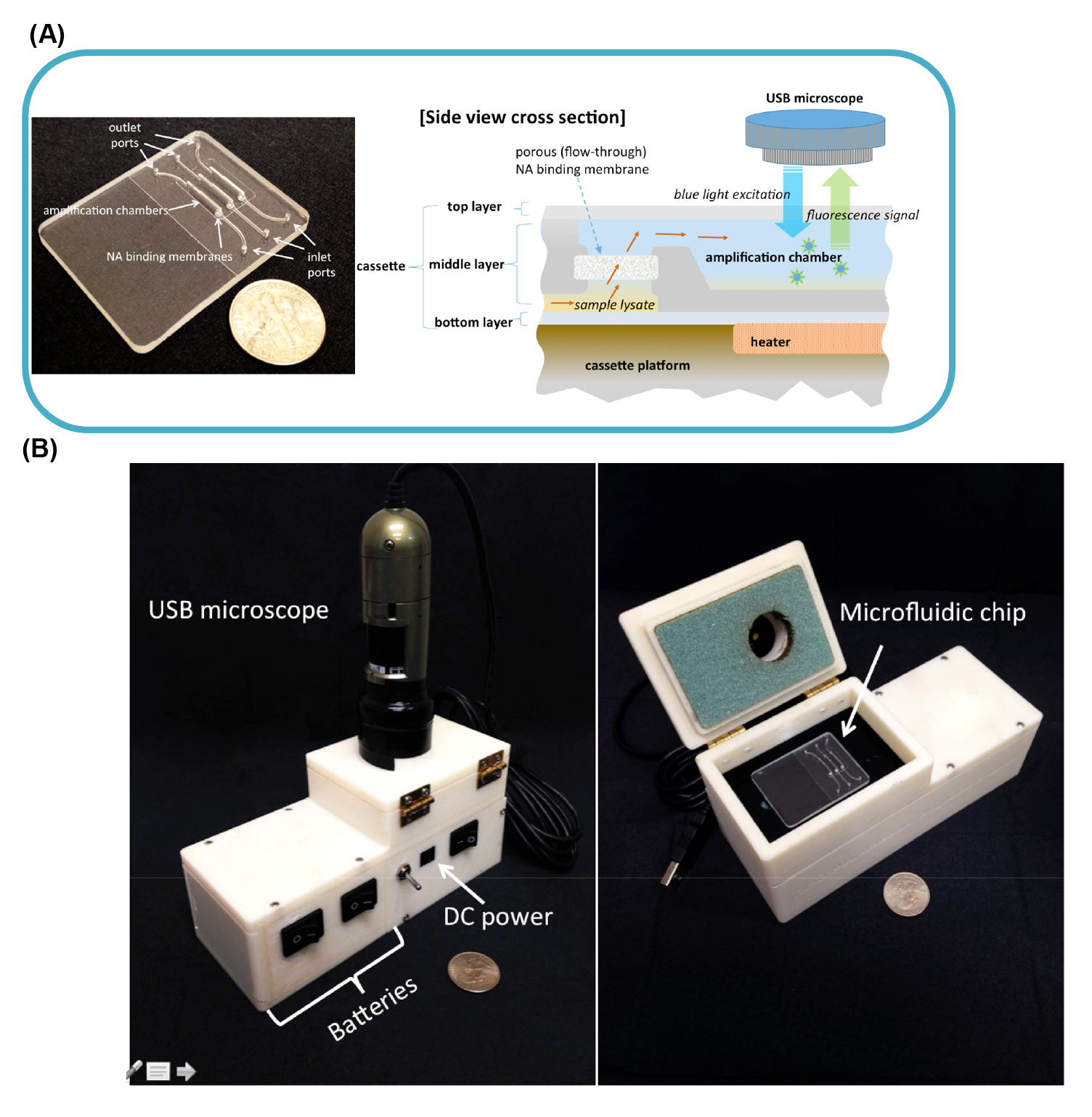

Supplement: S1 Fig — (A) A photograph and a schematic depiction of a POC chip with three reaction chambers. Nucleic acid capture, washing, amplification, and detection are all carried out in a single chamber that houses the nucleic acid binding membrane. (B) The custom-made, portable processor for nucleic acid isothermal amplification and detection that we used in our experiments. The USB microscope can be replaced with a smartphone camera. (TIF) [file pntd.0004318.s001.tif]

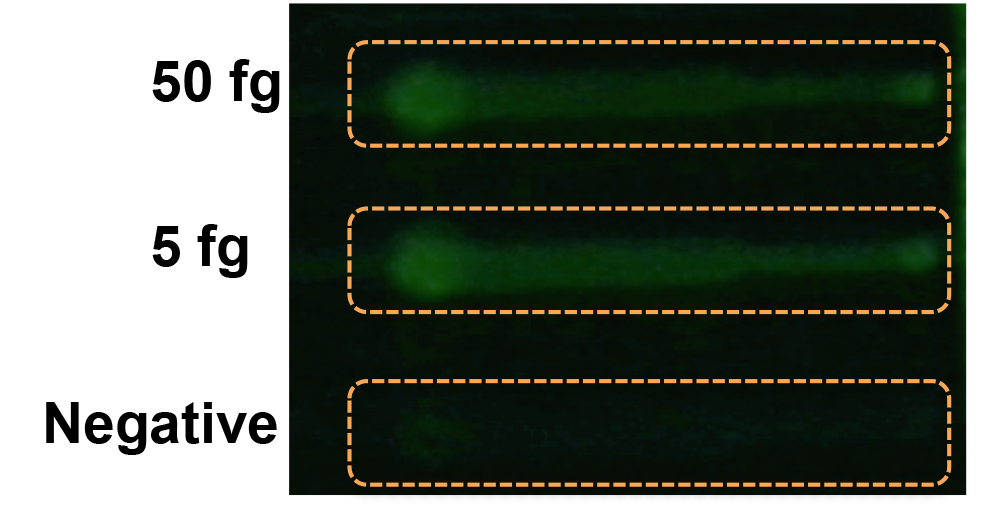

Supplement: S2 Fig — Images of fluorescence emission at t = 65 min from three reaction chambers during on-chip LAMP amplification of SM1-7 from 50 fg (top), 5 fg (middle), and 0 fg (no target control, bottom) S. mansoni gDNA. The image was taken using a Samsung Galaxy S3 smartphone. Excitation light source is from the flashlight of the smartphone with an added emission optical filter. Further experiment details are described in [44]. (TIF) [file pntd.0004318.s002.tif]

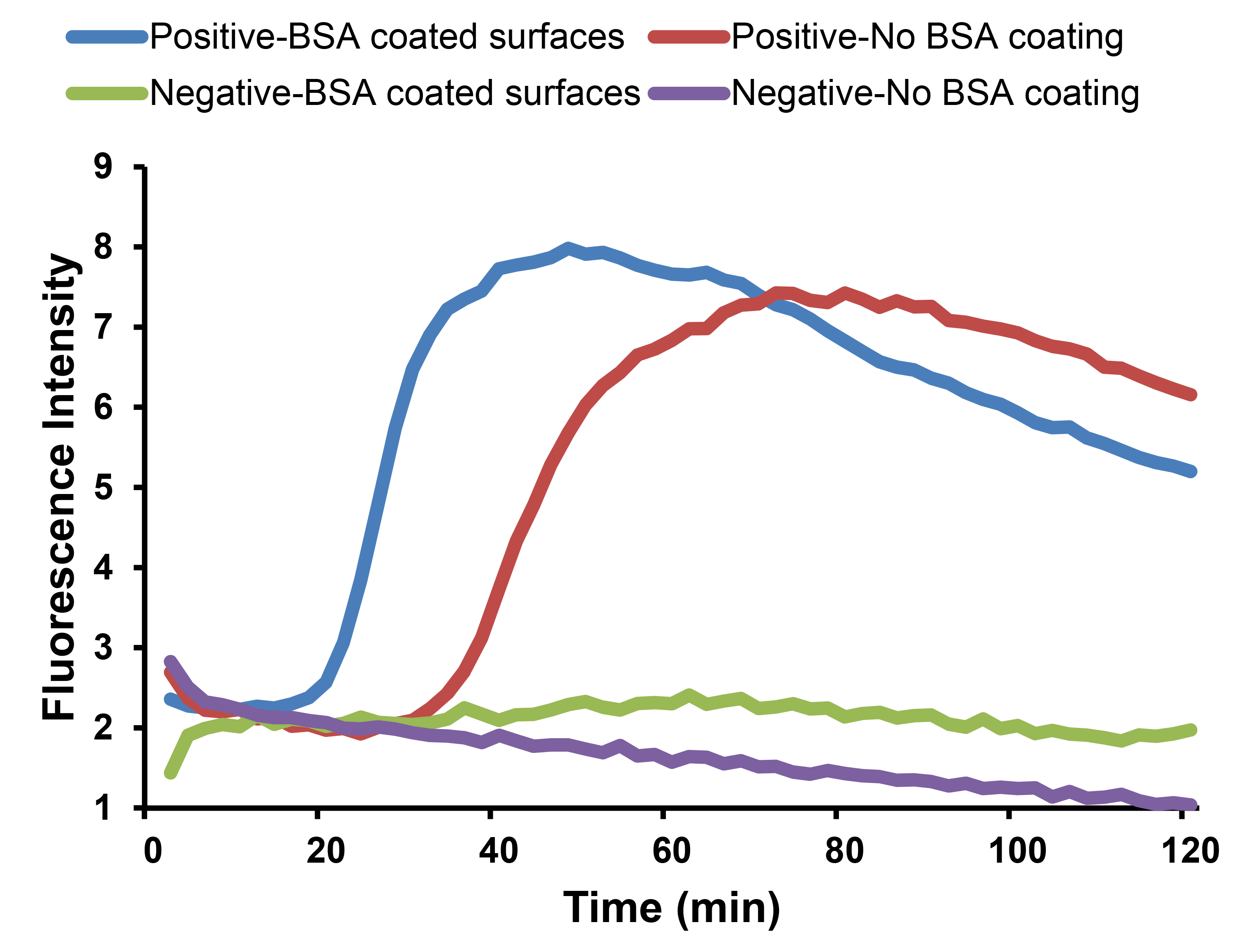

Supplement: S3 Fig — The chamber surface was coated with 10% BSA, left to dry overnight, and then washed. The chamber was then used to amplify 50 fg S. mansoni gDNA in serum. The threshold times in the BSA-coated and uncoated chambers were, respectively, 27 ± 1.4 and 41 ± 1.4 min (n = 2). We hypothesize that the BSA coating reduced enzyme unspecific binding to surfaces, thereby increasing amplification efficiency. The BSA-coated reactor performed similarly to the benchtop. (TIF) [file pntd.0004318.s003.tif]

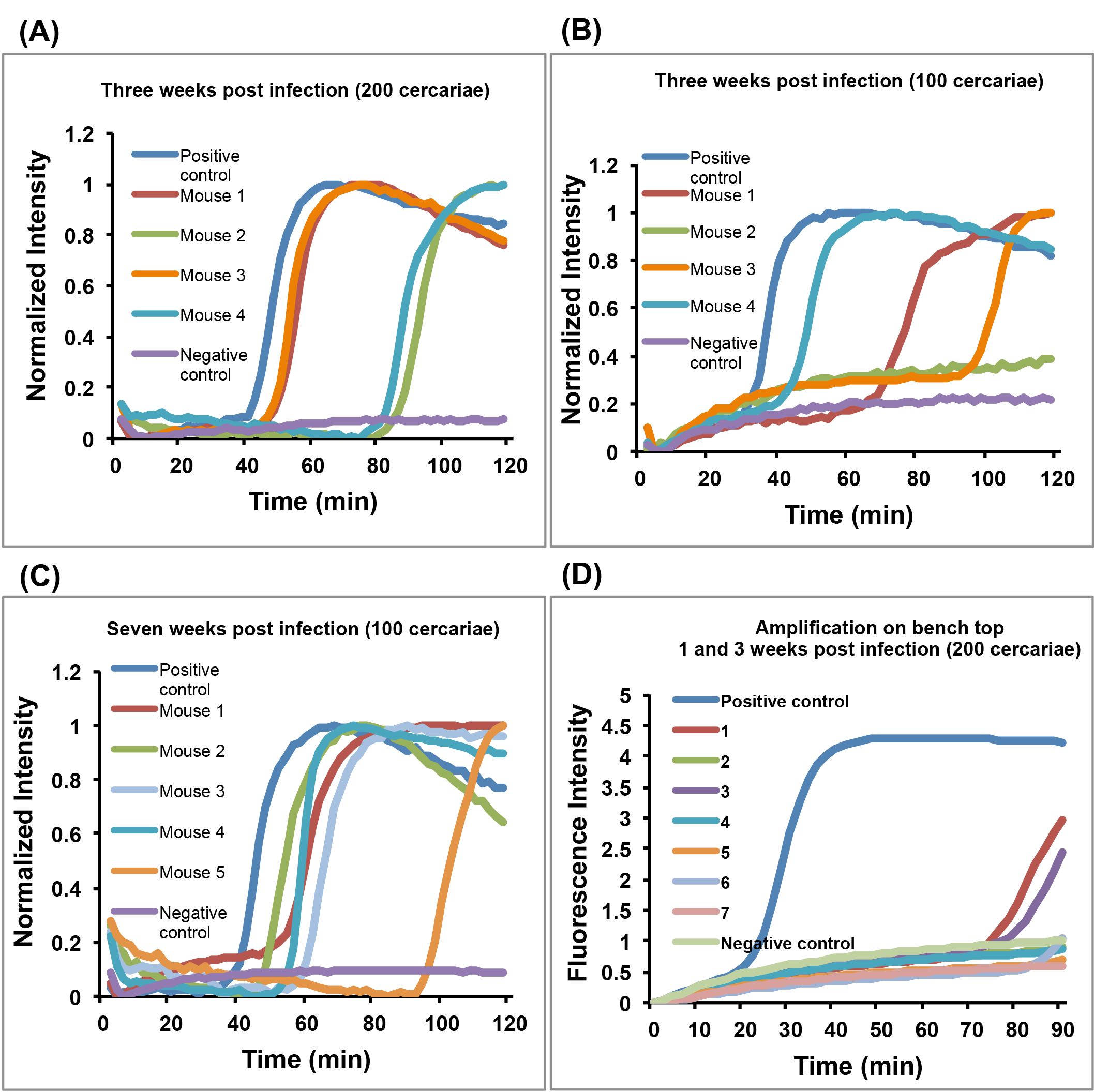

Supplement: S4 Fig — (A) Mice infected with ~200 cercariae, tested 3 weeks post infection. (B) Mice infected with ~100 cercariae, tested 3 weeks post infection. (C) Mice infected with ~100 cercariae tested 7 weeks post infection. The adult worm recovery number for mice 1, 2, 3, 4, and 5 in C were, respectively, 17, 49, 13, 22, and 4 parasites (adult worms were perfused from mice at ~7 weeks and counted). The positive control consists of 50 fg of S. mansoni gDNA spiked in 20 μL plasma. (D) Amplification on benchtop using 1 μL serum from the same mice as in (A) and Fig 4. Curves 1, 2, 3, and 4 correspond to 3-week-infected mice. Curves 5, 6, and 7 correspond to 1-week infected mice. The experimental conditions are the same as in Fig 3B. (TIF) [file pntd.0004318.s004.tif]
